# Books and Authors

**Published:** 1910-04

**Authors:** 


### BOOKS AND AUTHORS.

---

**International Clinics.** A Quarterly of Illustrated Clinical Lectures and especially prepared articles on Treatment, Medicine, Surgery, Neurology, Pediatrics, Obstetrics, Gynecology, Orthopedics, Pathology, Dermatology, Ophthalmology, Otology, Rhinology, Laryngology, Hygiene and other topics of interest to students and practitioners Edited by **W. T. Longcope**, M.D. Volume IV. Nineteenth series. Philadelphia and London. J. B. Lippincott Co. 1909. (Cloth, $2.00.)

Five articles on treatment embrace the following titles: antimeningitis serum and the results of its employment, by Simon Flexner; treatment of cancer by fulguration, by Pierre Fredet; use of tuberculin treatment, by Louis Hamman; general consideration of the therapeutic uses of diet, by George M. Niles; hypnotism, and psychotherapy, by J. W. Wainwright. The five articles on medicine are diagnosis and treatment of pernicious anemia, by Walter L. Bierring; adrenal insufficiency, by Emile Sergent; prognosis in advanced tuberculosis, by William B. Stanton; gouty phlebitis, by Sir Dyce Duckworth; duty of the physician in prevention of organic disease of the heart, by Henry M. Goodman. On surgery the topics are. fifteen cases operated upon in Professor Rodman's clinic at the Medico-Chirurgical Hospital, by J. Stewart Rodman; epiploitis following radical cure of hernia, by Charles Greene Cumston; color photographs in relation to surgery, by C. B. Longnecker; use of ethyl chloride as a general anesthetic, by W. Estell Lee. One article on röntgenology is entitled, the röntgen diagnosis of pulmonary tuberculosis, by Charles Lester Leonard.

The remaining articles are three on gynecology and obstetrics, one on genitourinary diseases, two on pediatrics, two on parasitology, one on laryngology, and two on pathology. John B. Deaver's article on the indications for surgery of the prostate is of the first importance. The entire book is of great interest.

---

**Transactions of the thirty-first annual meeting of the American Laryngological Association** held at Boston, May 31, June 1, and 2, 1909. **James E. Newcomb,** M.D., Secretary.

The president, Dr. Algernon Coolidge, Jr., of Boston, in his address dealt with topics of general interest to the Association, instead of choosing a scientific subject. Dr. W. Scott Renner, of Buffalo, presented a paper of much interest on "Nasal Tuberculosis," in which he gives the details of a case upon which he operated, removing a tumor which obstructed both nostrils. A joint session was held with the American Otological Association at which several papers were read relating to nasal and pharyngeal conditions as causative factors in aural disease. In addition to these the book contains many papers of much importance, all of which are of interest to laryngologists, and many of which were discussed in a spirited fashion.

---

**Transactions of the Section on Obstetrics and Diseases of Women** of the American Medical Association at the Sixtieth Annual Session, held at Atlantic City, N. J., June 8 to 11, 1909. **C. Jeff Miller,** M.D., New Orleans, Secretary.

The papers read at the meeting held at Atlantic City, June, 1909, in the Section on Obstetrics and Diseases of Women of the American Medical Association are bound in separate form and issued in this book. It is a splendid argument for the publication in book form of the transactions of a medical society. Dr. Walter P. Manton, of Detroit, was the chairman, and his address as such heads the list of papers and is entitled "Mental Alienation in Women and Abdomino-Pelvic Disease." Dr. Manton's large experience in dealing with nervous and mental diseases of women entitles him to speak interestingly on the subject and his address deserves reading by every gynecologist.

---

**Transactions of the American Dermatological Association.** Thirty-second annual meeting held at Annapolis, Md., September 24 and 25, and at Baltimore, September 26, 1908. **Grover W. Wende,** M.D., Buffalo, Secretary.

This association, one of the older of the special societies, held its annual meeting in 1908, two days in Annapolis and one day at Baltimore, under the presidency of Dr. Arthur Van Harlingen, of Philadelphia. The papers, always of high class, are quite up to their usual standard. The secretary, Dr. Grover W. Wende, presents a paper entitled "Keratosis follicularis resulting in epithelioma" during the course of which he reports a case, the patient having been presented before the American Association for cancer research in April and before the Alumnus Association Medical Department, University of Buffalo in May, 1908. It is elaborately illustrated by photographs of the patient, and photomicrographs of the pathological findings. The book is well printed, the discussions are excellent, but the binding is poor.

---

**Transactions of the American Urological Association.** Seventh annual meeting held at Chicago, June 1 and 2, 1908. Edited by **Charles Greene Cumston**, M.D.

This society publishes a handsome volume of transactions, and the papers it contains are excellent contributions to the literature of the specialty with which the association concerns itself. The president, Dr. Hugh H. Young insisted that his address be omitted, because of the fact that it was delivered extemporaneously, and illness prevented him from revising the proof. Bransford Lewis contributes a resume of progress in the development of the cystoscope, which is copiously illustrated and makes interesting reading. The discussions add much value to the papers, and a few are illustrated. The editor has done his part well in making a volume of much importance.

---

**Transactions of the American Surgical Association.** Vol. 27. Edited by **Richard H. Harte**, M.D., Recorder of the Association.

The president Dr. Charles B. G. de Nancrède, of Ann Arbor, in his address dealt principally with total excision of the scapula for sarcoma. The committee on necrology, presents reports on the deaths of William T. Bull, and Phineas S. Connor. The volume fairly bristles with important papers, foremost among which we may mention one by Rudolph Matas, entitled "The Fecal Origin of Some Forms of Postoperative Tetanus." Another paper of great value is by Albert Vander Veer entitled "Thoracic Surgery; Empyema." Professor Matas was elected president and the meeting this year will be held at Washington early in May.

---

**High Frequency Electric Currents in Medicine and Dentistry**; their nature, actions and simplified uses in external treatments. By **S. H. Monell**, M. D. Illustrated with special instruction plates. 8vo, 448 pages, extra cloth, price $4.00 net. New York: William R. Jenkins Company. 1910.

This has been called a wonder book of simple things. Whether this be so or not, interest begins in the first chapter, in which electricity and its peculiarities are defined. This particularly is conspicuous in the section, "Life Phenomena and Electricity," which tells what science has found out about the workings of nature in the human body, all set forth in an interesting manner. Then follow two chapters on Physiologic-Medical Properties of High-Frequency Currents, including a wonderful mass of so-called facts. And the section following these chapters concerning what others are doing with high-frequency currents will prove astonishing. Word pictures of treatment follow, and then twenty of the most absorbing chapters teaching in detail the advancement in treatment of various stages of diseases in which high-frequency currents can be made of benefit to patients. These twenty chapters are built on the physiologic foundation of the preceding sections. The book was written to assist the progressive surgeon, physician and dentist, and for all who have electricity in their homes. The use of high-frequency currents has been made in the household as well as in medicine and surgery.

---

**Transactions of the American Otological Society.** Forty-second annual meeting held at Harvard Medical School, Boston, June 1 and 2, 1909. Vol. XL. Part II. J. F. McKernon, M.D., Secretary.

The papers read at the forty-second annual meeting of this society held at the Harvard Medical School, Boston, June 1 and 2, 1910, are published in this book. The subjects dealt with are of importance and are handled by some of the eminent otologists of the land, including such men as Dench, Kyle, Halsted, Duel, Wright, and Richardson, besides many others we cannot find space or time to accord specific mention. Fifty-one members were present at this meeting which indicates a prosperous society. Among those in attendance we notice the name of Dr. W. Scott Renner, of Buffalo, whose reputation as a skillful otologist extends throughout the country.

**The Propaganda for Reform in Proprietary Medicines.** Sixth Edition. Containing the various exposes of nostrums and quackery which have appeared in the Journal of the American Medical Association. Pp. 292. Illustrated. (Paper, 10 cents; cloth, 35 cents.)

The council on pharmacy and chemistry of the American Medical Association puts out annually this "propaganda." This is the edition for 1910.

---

**Handbook of Therapy.** Pp. 421. Chicago: American Medical Association, 1910. (Cloth, $1.50.)

This handbook contains many useful hints on therapeutics which are stated clearly and concisely, making the book of convenient reference at the bedside. The articles have appeared in the *Journal of the American Medical Association*. Unusual drugs have been avoided and the formulas for the most part can be compounded by any pharmacist.

Besides the articles on therapy, the book contains a list of the articles accepted by the Council on Pharmacy and Chemistry for inclusion in new and nonofficial remedies, as well as tables and compilations of miscellaneous data. The book is of convenient size for the pocket or the satchel.

